# Inhibitory Control Mediates the Association between Perceived Stress and Secure Relationship Quality

**DOI:** 10.3389/fpsyg.2018.00217

**Published:** 2018-02-26

**Authors:** Toria Herd, Mengjiao Li, Dominique Maciejewski, Jacob Lee, Kirby Deater-Deckard, Brooks King-Casas, Jungmeen Kim-Spoon

**Affiliations:** ^1^Department of Psychology, Virginia Tech, Blacksburg, VA, United States; ^2^Department of Psychological & Brain Sciences, University of Massachusetts, Amherst, MA, United States; ^3^Department of Psychiatry, Amsterdam Public Health Research Institute, VU University Medical Center and GGZ inGeest, Amsterdam, Netherlands; ^4^Virginia Tech Carilion Research Institute, Roanoke, VA, United States

**Keywords:** adolescence, inhibitory control, perceived stress, relationship quality, functional magnetic resonance imaging

## Abstract

Past research has demonstrated negative associations between exposure to stressors and quality of interpersonal relationships among children and adolescents. Nevertheless, underlying mechanisms of this association remain unclear. Chronic stress has been shown to disrupt prefrontal functioning in the brain, including inhibitory control abilities, and evidence is accumulating that inhibitory control may play an important role in secure interpersonal relationship quality, including peer problems and social competence. In this prospective longitudinal study, we examine whether changes in inhibitory control, measured at both behavioral and neural levels, mediate the association between stress and changes in secure relationship quality with parents and peers. The sample included 167 adolescents (53% males) who were first recruited at age 13 or 14 years and assessed annually three times. Adolescents’ inhibitory control was measured by their behavioral performance and brain activities, and adolescents self-reported perceived stress levels and relationship quality with mothers, fathers, and peers. Results suggest that behavioral inhibitory control mediates the association between perceived stress and adolescent’s secure relationship quality with their mothers and fathers, but not their peers. In contrast, given that stress was not significantly correlated with neural inhibitory control, we did not further test the mediation path. Our results highlight the role of inhibitory control as a process through which stressful life experiences are related to impaired secure relationship quality between adolescents and their mothers and fathers.

## Introduction

Though adolescence can be a difficult transitional period in which risks to both physical and mental health, including depression, substance abuse, and suicide increase (Dahl, 2004), adolescents who have secure relationships with both their parents and peers may be able to navigate this period more successfully (Nickerson and Nagle, 2005). However, chronic stress has been implicated as a risk factor for interpersonal challenges. Past research has demonstrated negative associations between exposure to stressors and quality of interpersonal relationships, including parent–child relationships and peer relationships, among children and adolescents (Bonn, 1995; Mohr, 2006; Brown and Fite, 2016; Platt et al., 2016). Nevertheless, underlying mechanisms of this association remain unclear. Based on the neuroscience literature illustrating stress effects on prefrontal functioning and the developmental psychology literature indicating important roles of self-regulation in social development, we examined inhibitory control as a potential mediator in the association between stress and secure relationship quality.

Extant research has shown that children and adolescents with stressful life experiences tend to have negative relational experiences—with peers as well as parents. With respect to peer relationships, children who have been rejected by their peers are more likely than non-rejected children to have been exposed to multiple chronic stressors, including financial strain, living with a single parent, violence in the home, parental divorce, and family illness (Bonn, 1995; Baldry, 2003; Mohr, 2006). Furthermore, these stressors seem to create cumulative risk for adverse peer relations, suggesting that these difficulties are visible to children’s peer groups and predictive of adjustment difficulties within that peer group (Bonn, 1995). With respect to parent–child relationships, in one available study conducted to assess potential mechanisms in the association between stressful life events and child anxiety, a dysfunctional parent–child relationship emerged as a significant mediator, demonstrating a link whereby stressful life events may increase a parent’s self-reported dissatisfaction with their parent–child relationship (Platt et al., 2016). Another study demonstrated that the activation of stress hormones, including cortisol, predicted adolescents’ perception of problems within the family unit (Marceau et al., 2012). Finally, studies examining stressful life events on attachment outcomes indicate that caregiver stressful life events (e.g., abuse, neglect, divorce, caregiver death, and caregiver substance use) are associated with changes in attachment style over time (Waters et al., 2000; Weinfield et al., 2000).

To date, how stressful life experiences may be related to interpersonal relationships is not clearly understood. Mohr (2006) posits that the association between stress and interpersonal relationship quality may be due to the behavior exhibited by the children and youth experiencing these high degrees of family adversity. Often times, these individuals have difficulties modulating their own behavior and affect in interpersonal relationships. Unregulated, impulsive, and even perhaps aggressive behavior as a result may provoke negative interpersonal relationships. Similarly, research has suggested that the association between stressful life events and relationship quality may develop through social functioning deficits through an inappropriate pattern of relating to others and an inability to self-regulate (Brown and Fite, 2016).

Within the neuroscience literature, previous research has demonstrated that stress is associated with impaired structure and function of the prefrontal cortex (PFC), a brain region that contributes to self-regulation capabilities (see Hermans et al., 2014 for a review). Given that adolescence is a prolonged period of brain development, the immaturity and plasticity associated with this continued development, while adaptive in nature, leaves the brain vulnerable to potentially detrimental effects of enduring stress (Tottenham and Galván, 2016). While stress has deleterious effects on many brain regions (i.e., hippocampus, amygdala, see McEwen, 2007 for a review), given the present study’s focus on inhibitory control using the multi-source interference task (MSIT), we chose to restrict our review of the detrimental effects of stress on the brain to the PFC. Literature has shown the PFC to be the primary brain region related to self-regulation abilities, including cognitive inhibitory control and performance on the MSIT (Bush et al., 2003; Koechlin et al., 2003; Hermans et al., 2014). In rodent studies, chronic stress has been shown to alter the neuronal networks in the PFC by reducing dendritic length, branching, and spinal density (Arnsten, 2009). In a study designed to assess comparable changes in human adults experiencing stress, functional imaging data revealed that executive functions engage the PFC network and that the disruption of the integrity and connectivity of the PFC network results in impaired executive function abilities (Liston et al., 2009). Though caution must be taken in generalizing these results from rodents to humans, the disruption of the frontoparietal attention network in this study seems consistent with the demonstration in rodent studies of significant alterations to dendrites that not only impair connections within the PFC but also with surrounding areas (Liston et al., 2009). As a result, these structural changes may have important implications for the functioning of the PFC as a top-down regulatory system as well as the functional coupling between structures in these networks. Furthermore, the effects of stress on the PFC may be particularly harmful for developing brains. Indeed, in a study examining stress effects on cognitive control performance and related prefrontal functioning, although both adolescents and adults showed impaired inhibitory control performance, adolescents exhibited less recruitment of the dorsolateral prefrontal cortex (DLPFC) during inhibition under high stress versus low stress, whereas adults showed the opposite activation pattern in the DLPFC (Rahdar and Galván, 2014). In sum, current literature suggests that brain regions associated with the development of cognitive control in childhood and adolescence are generally affected by stress (see Arnsten, 2009 for a review). Particularly, in adolescents, stress-related dopamine release might flood an already saturated dopaminergic system to risk and reward. Excess dopamine receptor binding in the adolescent PFC then could lead to compromised PFC function, further subverting maturing regulatory functions (Uy and Galván, 2017).

Recent research is beginning to clarify the effects of these structural changes due to stress, suggesting impairments in executive functions, including in inhibitory control, attention, and memory. For example, children exposed to chronic stress via poverty display multiple self-regulatory deficits. In a behavioral study, children who experienced chronic poverty were rated by both parents and teachers as weaker in inhibitory control and delaying gratification (Evans and Kim, 2012). Similarly, in a functional magnetic resonance imaging study, adolescents who had experienced early life stress displayed impaired inhibitory control, as demonstrated by both poorer behavioral performance and greater activation in brain regions associated with inhibitory control (Mueller et al., 2010). Such results are consistent with findings using an adult sample demonstrating that those experiencing chronic stress exhibited cognitive deficits on a number of tasks requiring executive functions, including inhibitory control (Arnsten, 2009). Although correlational, taken together, previous findings suggest that there may be neurocognitive consequences of exposure to chronic stress.

Such consequences may manifest themselves within the quality of interpersonal relationships. For instance, Farley and Kim-Spoon (2014) emphasized the impact of self-regulation on interpersonal relationships during adolescence with individuals who were better able to self-control demonstrating higher quality relationships with parents and peers. Indeed, the literature demonstrates a robust link between inhibitory control and interpersonal relationship quality, suggesting that inhibitory control is necessary for successful interpersonal relationships. In early childhood, children who exhibit poorer inhibitory control at age 3 were more likely to have negative relations with peers at age 4 than those with better inhibitory control (Balaraman, 2003). Another study demonstrated that inhibitory control contributes to later social competence among preschoolers (Nigg et al., 1999). Further, in a recent longitudinal study spanning from early childhood to middle adolescence, poorer executive function abilities (including inhibitory control, working memory, and attention) increased the likelihood of peer problems later on (Holmes et al., 2016). These studies directly support the assertion that inhibitory control is related to peer relationship quality. Though no prior studies have explicitly tested the association between adolescent inhibitory control and parent–adolescent relationship quality, we draw on reviews suggesting that inhibitory control abilities are critical for developing positive relationships in general, encompassing parent–child, peer, friend, and romantic partner relationships (Farley and Kim-Spoon, 2014), and expect that adolescents with poor inhibitory control may experience difficulties in their relationships with mothers and fathers.

In the current longitudinal study, we aimed to investigate whether inhibitory control may be an explanatory process of the detrimental effects of stress on interpersonal relationships. Specifically, we hypothesized that earlier perceived stress is related to later secure interpersonal relationship quality over time via inhibitory control abilities (after controlling for baseline levels of the mediator and outcome). Past research thus far has primarily focused on objective indicators of stress (e.g., financial difficulties, familial violence; Bonn, 1995; Baldry, 2003; Mohr, 2006), thus limiting our understanding of the role of an individual’s subjective interpretation of stressors. According to the theoretical model proposed by Lazarus (1990), the experience of a stressor depends, at least in part, on the individual’s perception of how well they can control and manage it. Given that adolescence is a period in which stress tends to increase due to puberty, autonomy and identity formation, and relationship reorganization (Arnett, 1999; Tottenham and Galván, 2016), it is important to examine adolescents’ perceived stress as a risk factor related to the changing nature of their interpersonal relationships with parents and peers. The present study extends the literature in several significant ways. First, it focuses on individual perceptions of stress rather than objectively stressful events. Second, it examines the roles of both behavioral and neural indicators of adolescent inhibitory control as an explanatory mechanism in the association between perceived stress and secure relationship quality. Third, it examines potentially differential effects of stressful experiences on relationships with mothers, fathers, and peers. Finally, given sex differences in the brain, including volumetric differences in areas related to executive functions (including inhibitory control) and in interpersonal relationships that suggest that females are more sensitive to interpersonal cues (e.g., Rose and Rudolph, 2006; Gur and Gur, 2016), we also explored differences in the patterns of the associations among perceived stress, inhibitory control, and secure relationship quality between males and females.

## Materials and Methods

### Participants

The current community sample included 167 adolescents (53% males, 47% females) from southwestern Virginia, the United States of America. Adolescents were 13 or 14 years of age at Time 1 (*M* = 14.13, *SD* = 0.54), 14 or 15 years of age at Time 2 (*M* = 15.05, *SD* = 0.54), and 15 or 16 years of age at Time 3 (*M* = 16.07, *SD* = 0.56; data were collected between January 2014 and January 2017). Adolescents primarily identified as 80% Caucasian, 13% African–American, and 7% other. Mean family income was $25,000–$34,999 per year at Time 1 and Time 2 and $35,000–$49,999 per year at Time 3. At Time 1, 157 families participated. At Time 2, 10 families were added for a final sample of 167 parent–adolescent dyads. However, 24 families did not participate at all possible time points for reasons including: ineligibility for tasks (*n* = 2), declined participation (*n* = 17), and lost contact (*n* = 5) during the follow-up assessments. We performed attrition analyses using general linear model (GLM) univariate procedure to determine whether there were systematic predictors of missing data. Results indicated that rate of participation (indexed by proportion of years participated to years invited to participate) was not significantly predicted by demographic variables (*p* = 0.86 for age, *p* = 0.49 for income, *p* = 0.05 for sex, *p* = 0.20 for race, contrasted as White vs. non-White) or study variables at Time 1 (*p* = 0.40 for perceived stress, *p* = 0.49 for inhibitory control, *p* = 0.62 for mother relationship quality, *p* = 0.60 for father relationship quality, *p* = 0.87 for peer relationship quality).

### Procedures

Adolescent participants and their parents were recruited as part of a longitudinal study via email announcements, flyers, notice on the internet, or snowball sampling (word-of-mouth). The current study used data from Time 1, Time 2, and Time 3; each assessment was approximately 1 year apart from the previous one. Data collection took place at the university’s offices where adolescents and their primary caregivers were interviewed by trained research assistants. Both parents and adolescents received monetary compensation for their time. All procedures were approved by the institutional review board of the university with written informed consent and assent from all participants.

### Measures

#### Perceived Stress

Perceived stress was assessed using adolescent self-report at Time 1 using the 10-item perceived stress scale (PSS; Cohen and Williamson, 1988) that has been well validated to assess for perceptions of stress. Adolescents were asked to respond on a 5-point Likert scale from “0 = Never” to “4 = Very Often” about thoughts and feelings they’ve experienced within the past month. Sample items include, “In the last month, how often have you felt nervous and ‘stressed’?” and “In the last month, how often have you felt that you were on top of things?” (reverse scored). Mean scores were calculated across the 10 items from participants at Time 1, such that higher scores were indicative of higher perceived stress (α = 0.83).

#### Secure Relationship Quality

The short version of the Inventory of Parent and Peer Attachment was utilized at Time 1 and Time 3 to determine the degree of adolescents’ perceived security in their relationships with parents and peers (IPPA; Raja et al., 1992). Adolescents responded to three separate scales, each capturing a different relationship, including mother, father, and peers, using a 5-point Likert scale from “1 = Almost Never or Never True” to “5 = Almost Always or Always True.” Means scores were calculated for each relationship across the three subscales (communication, trust, and alienation; four items each) to create an overall attachment score, such that higher scores indicate higher levels of perceived secure relationship quality. Sample items include “I tell my mother/father about my problems and troubles” for the parent scales and “My friends encourage me to talk about my difficulties” for the peer scale (α = 0.78–0.92).

#### Inhibitory Control

At Time 1 and Time 2, adolescents’ inhibitory control was captured by the MSIT (Bush et al., 2003), using an in-house software application written in python using the VisionEgg (Straw, 2008) stimulus presentation library. In this task, participants were presented with sets of three numbers for duration of 1.75 s and asked to identify the number that differs from the other two. In the neutral condition, the distractor numbers were zeros, and the identity of the target was congruent with their position on the button box and screen. In the interference condition, the distractors were 1, 2, or 3 and the target’s identity was incongruent with its position on the button box and screen. Adolescents performed this task while their blood-oxygen-level-dependent (BOLD) response was monitored using functional magnetic resonance imaging. Participants completed 4 blocks of 24 neutral trials interleaved by 4 blocks of 24 interference trials for a total of 96 neutral trials and 96 interference trials. As with prior literature, we found a significant MSIT interference effect (i.e., main effect of congruency) in reaction time for correct responses, *t*(153) = 69.58, *p* < 0.001, as well as accuracy for correct responses, *t*(153) = -15.47, *p* < 0.001. These results showed that accuracy was lower and reaction time was higher (i.e., slower) for interference compared to neutral trials. A *behavioral* inhibitory control factor score was calculated using two indicators from this task: (1) the difference in accuracy of the neutral and interference trials (accuracy interference minus neutral) and (2) the intra-individual variability in reaction time or the intra-individual standard deviations across correct response latency trials in the interference condition. These two indicators were significantly correlated (*r* = -0.52, *p* < 0.001). Higher scores for the accuracy difference and lower scores for the intra-individual variability indicated better cognitive control. Confirmatory factor analysis (CFA) was performed to produce a factor score using standardized scores of these two indicators. At both waves, the models were fully saturated (χ^2^ = 0, *df* = 0, *p* = 0, CFI = 1.00) with significant loadings for the two indicators (Time 1: 0.72, *p* < 0.001; Time 2: 0.57, *p* < 0.001).

#### Imaging Acquisition and Analysis

Functional neuroimaging data were acquired on a 3T Siemens Tim Trio MRI scanner with a standard 12-channel head matrix coil. Echo-planar images (EPIs) were collected using the following parameters: slice thickness = 4 mm, 34 axial slices, field of view (FoV) = 220 mm × 220 mm, repetition time (TR) = 2 s, echo time (TE) = 30 ms, flip angle = 90 degrees, voxel size = 3.4375 mm × 3.4375 mm × 4 mm (during analysis the images were resliced so that voxels were 3 mm × 3 mm × 3 mm), 64 × 64 grid, and slices were hyperangulated at 30 degrees from anterior–posterior commissure. The structural scan was acquired using a high-resolution magnetization prepared rapid acquisition gradient echo sequence with the following parameters: TR = 1200 ms, TE = 3.02 ms, FoV = 245 mm × 245 mm, and 192 slices with the spatial resolution of 1 mm × 1 mm × 1 mm.

Data were pre-processed and analyzed using SPM8 (Wellcome Department of Imaging Neuroscience, London, United Kingdom). Functional images were corrected for head motion using a six-parameter rigid-body transformation, realigned, and normalized to a Montreal Neurological Institute (MNI) template using parameters derived from a segmented anatomical image coregistered to the mean EPI. The resulting image was spatially smoothed using an 8 mm full-width at half-maximum Gaussian kernel. Each participant’s preprocessed imaging data were whitened and analyzed using a GLM that included a boxcar regressor for each condition of interest, six motion-parameters as nuisance regressors, and a high-pass filter with cutoff at 128 s. Temporal autocorrelations were estimated using an autoregression AR(1). A subsequent second-level random effects analysis was conducted on individual interference minus neutral contrasts. Spherical regions-of-interest (ROI), 6 mm in radius and centered at peak voxels in the second-level analysis, were extracted using a family-wise corrected (FEW) threshold of *p* < 0.001.

Regions-of-interest values were considered based on (1) regions known to be engaged by inhibitory control related to interference and error processing (Koechlin et al., 2003; Roberts and Hall, 2008; Fitzgerald et al., 2010) and (2) regions significantly correlated with behavioral performance (i.e., absolute magnitude of correlation >0.2 with the behavioral performance factor score at each assessment). Seven ROIs in Time 1 (left posterior-medial frontal cortex, right inferior frontal gyrus, left and right inferior parietal lobules, right insula, right superior frontal gyrus, and left middle frontal gyrus) and three ROIs in Time 2 (left posterior-medial frontal cortex, left middle frontal gyrus, and left inferior frontal gyrus) met the criteria. Two of these ROIs emerged in both assessments (left posterior-medial frontal cortex, left middle frontal gyrus), and were chosen as manifest indicators of the *neural* inhibitory control factor scores created for Time 1 and Time 2. At each wave, CFA was performed to produce a factor score using standardized scores of two ROIs. At both waves, the models were fully saturated (χ^2^ = 0, *df* = 0, *p* = 0, CFI = 1.00) with significant loadings for two indicators (Time 1 factor loadings: 0.81, *p* < 0.001; Time 2 factor loadings: 0.77, *p* < 0.001).

### Plan of Analysis

For all study variables, descriptive statistics were examined to determine normality of distributions and outliers. Skewness and kurtosis were examined for all variable distributions and acceptable levels were skewness less than 3 and kurtosis less than 10 (Kline, 2011). Outliers were identified as values ≥3 *SD* from the mean. In these cases (*n* = 7), values were winsorized to retain statistical power and attenuate bias resulting from elimination (Ghosh and Vogt, 2012). Multivariate GLM analyses exhibited that demographic variables (adolescent age, gender, race, and family income) at Time 1 were not significant predictors of mother, father, or peer relationship quality at Time 3 (all *p*s > 0.10). Thus, they were not included as covariates in the main analyses.

The hypothesized model was tested via structural equation modeling (SEM) in MPlus 7.4 (Muthén and Muthén, 2012). The analyses followed recommendations for testing mediation models by Hayes (2013). To begin, we calculated a residualized change score for inhibitory control by regressing inhibitory control at Time 2 on inhibitory control at Time 1. We also calculated residualized change scores for relationship qualities with parents and peers by regressing each relationship quality at Time 3 on corresponding relationship quality at Time 1. The residualized change scores represent the change across time, and compared to simple difference scores, have the advantage that they adjust for baseline differences (MacKinnon et al., 2013).

The mediation model included paths (a) from perceived stress at Time 1 to changes in inhibitory control from Time 1 to Time 2, (b) from changes in inhibitory control to secure relationship quality with mother, father, and peers from Time 1 to Time 3, and (c) from perceived stress at Time 1 to changes in secure relationship quality with mother, father, and peers. We also estimated correlations among the three secure relationship quality outcomes. Overall model fit indices were determined by χ^2^ goodness of fit test, root mean square error of approximation (RMSEA), and confirmatory fit index (CFI). RMSEA values of less than 0.05 were considered a close fit while values less than 0.08 were considered a reasonable fit (Browne and Cudeck, 1993), and CFI values of greater than 0.90 were considered an acceptable fit while values greater than 0.95 were considered an excellent fit (Bentler, 1990). We calculated bias-corrected bootstrap confidence intervals (CIs) for the indirect effects using 10,000 bootstrapping samples (MacKinnon et al., 2004). These CIs take non-normality of the estimates into account and are therefore not necessarily symmetric (Muthén and Muthén, 2012). Full information maximum likelihood (FIML) estimation procedure (Arbuckle, 1996) was used for missing data since FIML estimates are superior to those obtained with listwise deletion or other *ad hoc* methods (Schafer and Graham, 2002). In order to test whether the regression paths were moderated by sex, we additionally ran a multiple group model and tested whether imposing equality constraints on the regression parameters between males and females degraded model fit significantly using the Wald test.

## Results

Correlations and descriptive statistics for all study variables can be found in **Table 1**. We first tested the effects of perceived stress at Time 1 on secure relationship quality for mothers, fathers, and peers at Time 3 after controlling for their respective relationship qualities at Time 1 (represented by residualized change scores) via *behavioral* inhibitory control at Time 2 after controlling for behavioral inhibitory control at Time 1 (represented by a residualized change score). The original model was a fully saturated model, with χ^2^ = 0, *df* = 0, *p* = 0, CFI = 1.00, RMSEA = 0. The direct effects from perceived stress at Time 1 to the secure mother relationship quality residualized change score (*b* = 0.10, *SE* = 0.07, *p* = 0.136) and secure father relationship quality residualized change score (*b* = 0.05, *SE* = 0.09, *p* = 0.604) were not significant. The effect from behavioral inhibitory control to the secure peer relationship quality residualized change score was also not significant (*b* = 0.02, *SE* = 0.07, *p* = 0.826). For model parsimony, we constrained these paths to zero.

**Table 1:** Descriptive statistics and bivariate correlations among perceived stress, behavioral and neural inhibitory control, and secure relationship quality.

	1	2	3	4	5	6	7	8	9	10	11	12	13	14	15	16
(1) Perceived stress T1															
(2) Behavioral IC T1	0.02														
(3) Behavioral IC T2	-0.20*	0.53*													
(4) Behavioral IC residualized change score (T2 on T1)	-0.27**	0.00	0.85**												
(5) Neural IC T1	0.08	-0.37**	-0.29**	-0.13											
(6) Neural IC T2	0.12	-0.23*	-0.26**	-0.14	0.36**										
(7) Neural IC residual score at T2 on T1	0.11	-0.15	-0.18	-0.12	0.00	0.93**									
(8) Mother relationship quality T1	-0.36**	-0.14	0.11	0.21*	0.00	-0.03	-0.09								
(9) Mother relationship quality T3	-0.24**	0.00	0.32**	0.41**	-0.12	-0.06	-0.05	0.62**							
(10) Mother relationship quality residualized change score (T3 on T1)	0.03	0.07	0.31**	0.34**	-0.12	-0.04	0.00	0.00	0.78**						
(11) Father relationship quality T1	-0.26**	-0.15	0.11	0.21*	0.01	-0.08	-0.11	0.47**	0.43**	0.23**					
(12) Father relationship quality T3	-0.28**	-0.06	0.27**	0.38**	-0.09	0.02	0.08	0.23*	0.43**	0.38**	0.71**				
(13) Father relationship quality residualized change score (T3 on T1)	-0.04	0.06	0.29**	0.31**	-0.06	0.10	0.19	-0.10	0.18*	0.32**	0.00	0.71**			
(14) Peer relationship quality T1	-0.27**	-0.13	0.06	0.15	-0.09	-0.01	0.00	0.36**	0.14	-0.11	0.20*	0.11	-0.07		
(15) Peer relationship quality T3	-0.26**	-0.07	0.09	0.14	-0.27**	-0.18	-0.09	0.31**	0.39**	0.25**	0.29**	0.28**	0.09	0.42**	
(16) Peer relationship quality residualized change score (T3 on T1)	-0.14	-0.02	0.06	0.07	-0.22*	-0.16	-0.09	0.18*	0.36**	0.32**	0.22*	0.25**	0.14	0.00	0.91**
*M*	1.48	0.00	0.00	0.00	0.00	0.01	0.00	4.04	3.96	0.00	3.84	3.61	0.00	3.99	4.10	0.00
*SD*	0.66	0.83	0.71	0.61	0.87	0.80	0.76	0.61	0.67	0.54	0.67	0.88	0.63	0.56	0.47	0.43

*T1 = Time 1; T2 = Time 2; T3 = Time 3. ^∗^p < 0.05, ^∗∗^p < 0.01, ^∗∗∗^p < 0.001.*

**FIGURE 1:**
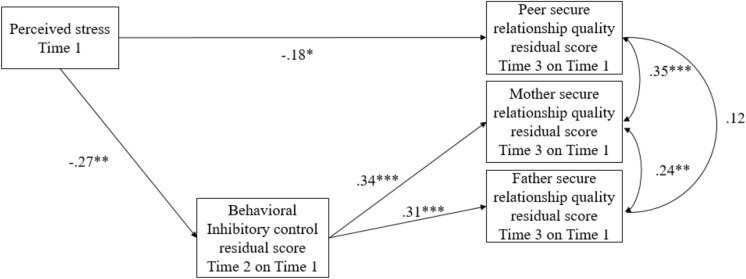
Standardized parameter estimates for the associations among perceived stress, the behavioral inhibitory control residualized change score, and secure relationship quality residualized change scores. ^∗^*p* < 0.05, ^∗∗^*p* < 0.01, ^∗∗∗^*p* < 0.001.

The final model showed an excellent fit, χ^2^ = 2.25, *df* = 3, *p* = 0.52, CFI = 1.00, RMSEA = 0 (see **Figure 1** for standardized coefficients). Higher levels of perceived stress at Time 1 were associated with lower inhibitory control residualized change scores (*b* = -0.25, *SE* = 0.08, *p* = 0.001). In turn, lower inhibitory control residualized change scores were related to lower secure relationship quality residualized change scores for adolescents and their mothers (*b* = 0.30, *SE* = 0.07, *p* < 0.001) and fathers (*b* = 0.32, *SE* = 0.09, *p <* 0.001). The indirect effects of perceived stress at Time 1 on the secure mother relationship quality residualized change score (*b* = -0.08*, SE* = 0.03, 95% CI [-0.160, -0.026], *b^∗^* = -0.09) and the secure father relationship quality residualized change score (*b* = -0.08*, SE* = 0.03, 95% CI [-0.159, -0.029], *b^∗^* = -0.08), through the behavioral inhibitory control residualized change score, were significant. For secure peer relationship quality, higher levels of perceived stress at Time 1 directly predicted a lower secure peer relationship quality residualized change score (*b* = -0.12, *SE* = 0.05, *p* = 0.03). Given that the behavioral inhibitory control residualized change score was not significantly associated with the secure peer relationship quality residualized change score, the indirect effect of perceived stress on the secure peer relationship quality residualized change score through the behavioral inhibitory control residualized change score was not estimated. In addition, the secure mother relationship quality residualized change score significantly correlated with that of the secure father relationship quality residualized change score (*b* = 0.07*, SE* = 0.03, *p* = 0.009) and the secure peer relationship quality residualized change score (*b* = 0.08*, SE* = 0.02, *p <* 0.001). However, the correlation between the secure father relationship quality residualized change score and the secure peer relationship quality residualized change score was not significant (*b* = 0.03*, SE* = 0.02, *p* = 0.174).

We then tested the effects of perceived stress at Time 1 on the secure relationship quality for mothers, fathers, and peers at Time 3 after controlling for their respective levels at Time 1 (represented by residualized change scores) via *neural* inhibitory control at Time 2 after controlling for neural inhibitory control at Time 1 (represented by a residualized change score). The original model was a fully saturated model, with χ^2^ = 0, *df* = 0, *p* = 0, CFI = 1.00, RMSEA = 0. Perceived stress at Time 1 did not predict the neural inhibitory control residualized change score (*b* = 0.16, *SE* = 0.11, *p* = 0.145, *b^∗^* = 0.14) or the three secure relationship quality residualized change scores (mother: *b* = 0.03, *SE* = 0.07, *p* = 0.691, *b^∗^* = 0.04; father: *b* = -0.08, *SE* = 0.09, *p* = 0.390, *b^∗^* = -0.08; peer: *b* = -0.08, *SE* = 0.06, *p* = 0.16, *b^∗^* = -0.13). The predictive effects of the neural inhibitory control residualized change score was only significant for the secure father relationship quality residualized change score (*b* = 0.19, *SE* = 0.08, *p* = 0.025, *b^∗^* = 0.23), but not for the secure mother relationship quality residualized change score (*b* = 0.003, *SE* = 0.08, *p* = 0.973, *b^∗^* = 0.004) or the secure peer relationship quality residualized change score (*b* = -0.06, *SE* = 0.06, *p* = 0.272, *b^∗^* = -0.11). Given that perceived stress at Time 1 was not related to the neural inhibitory control residualized change score, we did not further test the indirect effects of perceived stress at Time 1 on the three secure relationship quality residualized change scores via the neural inhibitory control residualized change score. In this model, the secure mother relationship quality residualized change score significantly correlated with that of the secure father relationship quality residualized change score (*b* = 0.11*, SE* = 0.03, *p* = 0.001, *b^∗^* = 0.32) and the secure peer relationship quality residualized change score (*b* = 0.08*, SE* = 0.02, *p <* 0.001, *b^∗^* = 0.33). However, the residual covariance between the secure father relationship quality residualized change score and the secure peer relationship quality residualized change score was not significant (*b* = 0.04*, SE* = 0.02, *p* = 0.105, *b^∗^* = 0.15).

To test whether the associations among perceived stress, inhibitory control, and secure relationship quality may vary by sex, we performed an omnibus multiple groups Wald test for behavioral inhibitory control model and neural inhibitory control model, respectively. The Wald test compared all regression paths between males and females in the original saturated model. In both tests, multiple groups analyses did not reveal any significant sex differences: Wald χ^2^(10) = 9.31, *p* = 0.503 for behavioral inhibitory control model and Wald χ^2^(10) = 7.50, *p* = 0.677 for neural inhibitory control model. Therefore, results suggested that the associations among perceived stress, behavioral/neural inhibitory control, and secure relationship quality were comparable between males and females.

### Supplemental Analyses

We reran the models with level scores (instead of residualized change scores) and tested the effects of Time 1 perceived stress on Time 3 secure relationship quality with mother, father, and peers via Time 2 behavioral/neural inhibitory control. Overall, the results on the level of these variables are highly similar to our original results using residualized change scores, confirming that the results of the residualized change score model are robust. Detailed results for behavioral/neural inhibitory control follow.

For *behavioral* inhibitory control, the original model was a fully saturated model, with χ^2^ = 0, *df* = 0, *p* = 0, CFI = 1.00, RMSEA = 0. The effect from behavioral inhibitory control to secure peer relationship quality was not significant (*b* = 0.03, *SE* = 0.06, *p* = 0.637, *b^∗^* = 0.04). For model parsimony, we constrained this path to zero. The final model showed an excellent fit, with χ^2^ = 0.22, *df* = 1, *p* = 0.64, CFI = 1.00, RMSEA = 0. Higher levels of perceived stress at Time 1 were associated with lower inhibitory control at Time 2 (*b* = -0.21, *SE* = 0.01, *p* = 0.016, *b^∗^* = *-*0.20). In turn, lower inhibitory control was related to lower secure relationship quality for adolescents and their mothers (*b* = 0.27, *SE* = 0.07, *p* < 0.001, *b^∗^* = 0.28) and fathers (*b* = 0.25, *SE* = 0.10, *p* = 0.012, *b^∗^* = 0.20) at Time 3. The indirect effects of perceived stress on secure mother relationship quality (*b* = -0.06*, SE* = 0.03, 95% CI [-0.144, -0.007], *b^∗^* = *-*0.06) and secure father relationship quality (*b* = *-*0.05*, SE* = 0.03, 95% CI [-0.134, -0.007], *b^∗^* = *-*0.04), through behavioral inhibitory control, were significant. For secure peer relationship quality, higher levels of perceived stress at Time 1 directly predicted lower secure peer relationship quality (*b* = -0.18, *SE* = 0.06, *p* = 0.002, *b^∗^* = *-*0.25). Given that behavioral inhibitory control was not significantly associated with secure peer relationship quality, the indirect effect of perceived stress at Time 1 on secure peer relationship quality at Time 3 through behavioral inhibitory control at Time 2 was not estimated.

We then tested the effects of perceived stress at Time 1 on secure relationship quality for mothers, fathers, and peers at Time 3 via *neural* inhibitory control at Time 2. The original model was a fully saturated model, with χ^2^ = 0, *df* = 0, *p* = 0, CFI = 1.00, RMSEA = 0. Perceived stress at Time 1 did not predict neural inhibitory control (*b* = 0.04, *SE* = 0.03, *p* = 0.194, *b^∗^* = 0.19), however, it predicted significant lower secure relationship quality with mothers (*b* = -0.24, *SE* = 0.085, *p* = 0.005, *b^∗^* = -0.23), fathers (*b* = -0.37, *SE* = 0.115, *p* = 0.001, *b^∗^* = *-*0.28), and peers (*b* = -0.17, *SE* = 0.059, *p* = 0.005, *b^∗^* = -0.23). The predictive effects of neural inhibitory control were not significant for secure mother relationship quality (*b* = -0.008, *SE* = 0.32, *p* = 0.979, *b^∗^* = *-*0.003), secure father relationship quality (*b* = 0.29, *SE* = 0.39, *p* = 0.455, *b^∗^* = 0.071), or secure peer relationship quality (*b* = -0.36, *SE* = 0.20, *p* = 0.067, *b^∗^* = *-*0.17). Given that perceived stress at Time 1 was not related to neural inhibitory control at Time 2, we did not further test the indirect effects of perceived stress at Time 1 on secure relationship quality at Time 3 via the neural inhibitory control at Time 2.

## Discussion

The present study sought to elucidate the underlying role of inhibitory control in explaining the association between stress and secure interpersonal relationship quality in adolesence. Results demonstrated indirect paths via adolescent behavioral inhibitory control such that perceived stress was related to lower inhibitory control which in turn was related to lower secure relationship quality between adolecents and their mothers and fathers. These findings were obtained despite controlling for baseline levels of inhibitory control and secure relationship quality. Our data suggested a direct link between adolescents’ perceived stress and their secure relationship quality with peers, such that higher perceived stress predicted poorer secure relationship quality with peers; however, the effects of stress on secure peer relationship quality was not mediated by behavioral inhibitory control. In contrast to the findings of behavioral inhibitory control, stress was not significantly correlated with neural inhibitory control. Thus, we did not test the indirect effects of perceived stress on secure relationship quality via neural inhibitory control. Finally, the pattern of findings was not moderated by sex, indicating that the associations among stress, behavioral and neural inhibitory control, and secure relationship quality were comparable between males and females.

The indirect effect of perceived stress on adolecent’s secure mother and father relationship quality via behavioral inhibitory control supported our hypothesis. In line with previous research in adults indicating an association between stress and impaired executive functioning related to self-regulation capabilities (Scholz et al., 2009; Hermans et al., 2014), our data indicated that adolescents who reported high levels of stress at an earlier time exhibited poor inhibitory control performance over time. In contrast to prior work using acute, experimentally manipulated stress, our measure of perceived stress assessed subjective evaluations of naturally occurring stress—which are thought to be more severe and ecologically valid than laboratory induced stress (Starcke and Brand, 2012). Our findings also expand prior findings by demonstrating the effects of stress on longitudinal changes in inhibitory control. Further, our results were supportive of prior work proposing that individuals with better self-regulation abilities have higher interpersonal relationship quality during adolescence (Farley and Kim-Spoon, 2014). Likely, adolescents with poorer inhibitory control display inappropriate, dysregulated behavior that leads to poorer interpersonal relationship quality (Brown and Fite, 2016).

Based on previous literature emphasizing the importance of self-regulation on interpersonal relationship quality (Farley and Kim-Spoon, 2014), we expected to find indirect paths from perceived stress to all three types of secure interpersonal relationships in adolescence (i.e., mother, father, and peer) through inhibitory control. However, differences in the nature of adolescent relationships with parents versus peers may explain why indirect effects through behavioral inhibitory control were significant for secure mother and father relationship quality but not for secure peer relationship quality. Although it is often thought that adolescents’ reliance on their parents for support decreases during this period, the literature has shown this is not necessarily the case. While peer relationships become more prominent in adolescence, parents have been and continue to be a source of support for many adolescents, indicating the continual primary attachment relationship during this period (Lieberman et al., 1999; Nickerson and Nagle, 2005). That is, parents do serve as important attachment figures throughout childhood and adolescence. The distinct differences in adolescents’ relationships with their parents versus their peers may suggest that inhibitory control, a within-person characteristic that is stable across time and context (Nigg, 2017), matters more for parent–adolescent relationships that involve more stable and intense interactions with relative permanance, compared to peer relationships that are everchanging, intentional, and relatively transient (Brown and Larson, 2009), partly due to the fact that adolescents often choose peers who like and accept them based on similarity (Veenstra and Dijkstra, 2011).

Our results demonstrating that inhibitory control was not directly associated with secure peer relationship qualilty appear to be inconsistent with previous research showing significant links between executive functions (including inhibitory control) and peer problems in early childhood through middle adolesence (Holmes et al., 2016). However, it is important to note that the predictive path between executive functions and peer problems decreased from 4.5 to 15 years of age in that study, indicating that executive functions became less important or less predictive of peer problems in adolescence as opposed to childhood (Holmes et al., 2016). One plausible explanation for this trend is the change in the nature of peer relationships from childhood to adolescence (Rubin et al., 1998). Adolescent peer relationships tend to increasingly value communication and disclosure which inhibitory control may not be as relevant to and therefore may not be the best predictor of secure peer relationship quality in adolescence (Holmes et al., 2016). A second explanation for the discrepency between our results and the findings by Holmes et al. (2016) may be the difference in the peer construct. The present study focused on secure relationship quality as indicated by perceived support and closeness, whereas the study by Holmes et al. (2016) focused on peer problems as indicated by peer rejection and victimization. Taken together, these findings suggest that poor inhibitory control may play an important role in predicting more extreme forms of difficulties in peer relationships, such as victimization rather than security of the relationship. Nonetheless, this result was not anticipated and thus requires further examination.

We found a significant direct link between perceived stress and secure relationship quality with peers. While no prior research to our knowledge has focused on perceived stress, objective stressful life events have been documented in the literature as a risk factor for negative peer relationships (Bonn, 1995; Baldry, 2003; Mohr, 2006). Thus, our results add to the extant literature as evidence for the link between perceived stress and secure peer relationship quality. Though future studies would benefit from testing additional mediators (e.g., emotion regulation, Kim and Cicchetti, 2010) that may explain the effects of perceived stress on peer relationship quality.

Our data suggested that, given the lack of significant association between stress and neural inhibitory control, the neural correlates of inhibitory control would not mediate the association between perceived stress and security in adolescent interpersonal relationships. The weak association between perceived stress and neural inhibitory control appears to be inconsistent with the neurosceince literature documenting the negative effects of stress on prefrontal functioning (Liston et al., 2009; Mueller et al., 2010). However, the nature of the stressful experience may be critical to understanding its relation to nerual processes underlying inhibitory control. For example, the present study found *behavioral* inhibitory control to be more strongly related to general perceived stress than neural inhibitory control. Prior studies involving adopted or foster children and adolescents with early-life stress defined by neglectful and abusive care revealed that these children showed impairements in neural inhibitory control compared to those who did not experience such early adversity (Mueller et al., 2010; Bruce et al., 2013). How different types of stressful experiences (e.g., caregiving adversity versus socioeconomic adversity) may be differentially related to brain development during adolescence warrants further investigation.

The present findings should be interpreted in light of several limitations. The outcome variable was self-reported by a single informant. Future studies should consider using multiple informants and multiple methods to reduce possible bias due to using a single informant. Additionally, although we used longitudinal data, the nature of correlational data prevent us from inferring causality. Moreover, future longitudinal research is recommended to test potential bidirectional effects among perceived stress, inhibitory control, and secure interpersonal relationship quality. For example, secure attachment relationships may promote adaptive responses to stress (e.g., Pierrehumbert et al., 2009) and inhibitory control (e.g., Bernier et al., 2012). At the same time, stress has the ability to deterioriate relationships (Bonn, 1995; Baldry, 2003; Mohr, 2006; Marceau et al., 2012; Platt et al., 2016). Thus, further research examining potential bidirectional effects between stress and secure relationship quality as they relate to inhibitory control is needed. The current investigation focused on mediated relationships between stressful life events and secure relationship quality via inhibitory control. Fruitful extensions for future research may include testing the role of inhibitory conrol in the stress-buffering hypothesis, which posits that attachment relationships have the ability to dampen the negative effects of stress on well-being (e.g., Hanson and Chen, 2010; Miller et al., 2016) and considering internal working models of attachment as an additional possible mediator in this association between stress and secure relationship quality (e.g., Collins and Feeney, 2004).

Furthermore, it will be particularly important to consider the bidirectional nature of the brain and environmental contexts (such as stressful life events and secure interpersonal relationships) within adolesence, given that stress exposures during adolescence can have more potent effects on the brain than when those exposures occur in adulthood – due to rapid brain development and increased plasticity of developing systems relative to adulthood (Tottenham and Galván, 2016). From a methodological viewpoint, it may be that selective weakening and strengthening of functional connectivity within cognitive control circuits (e.g., Botvinick et al., 2001) are the mechanism by which behavioral inhibition is achieved, whereas behavioral inhibition is the goal that mechanism is intended to achieve. Then, the BOLD responses in specific ROIs may not be effectively capturing such a mechanism. For example, evidence from research using task-based functional connectivity revealed the beneficial contributions of increased activation coherence within the cognitive control system (i.e., functional connectivity between the ventromedial PFC and dorsolateral prefrontal and parietal cortices) to less impulsive decision making among adolescents (Christakou et al., 2011).

Past research has rarely investigated the link between inhibitory control and positive developmental outcomes such as secure interpersonal relationship quality. Our longitudinal analyses suggest that adolescents with poor behavioral inhibitory control are likely to show poor secure relationship quality with both mothers and fathers over time. This association may be expected given that flexible inhibitory control promotes social affective skills (Crone and Dahl, 2012), ultimately enhancing interpersonal interaction quality. Aside from the direct association between stress and interpersonal relationships as found in prior studies (e.g., Bonn, 1995; Baldry, 2003; Mohr, 2006; Platt et al., 2016), the current study clarifies that perceived stress has detrimental effects on the behavioral manifestion of inhibitory control, and in turn is related to poorer secure relationship quality with parents.

## Conclusion

The identification of inhibitory control as a mechanism in the stress-secure interpersonal relationship quality association is beneficial in how it may inform intervention work for children and adolescents who are especially prone to chronic stress. Interventions that teach and practice skills related to inhibitory control may help protect against negative parent–adolescent relationships later on.

## Ethics Statement

This study was carried out in accordance with the recommendations of Virginia Tech Institutional Review Board for the Protection of Human Subjects, Office of Research Compliance with written informed consent from all subjects. All subjects gave written informed consent in accordance with the Declaration of Helsinki. The protocol was approved by the Virginia Tech Institutional Review Board for the Protection of Human Subjects.

## Author Contributions

TH conceived the study, participated in data collection, participated in statistical analyses and interpretation of the data, and drafted the manuscript. ML performed statistical analyses and interpretation of the data and drafted the manuscript. DM performed statistical analyses and interpretation of the data and critically revised the manuscript. JL participated in statistical analyses and interpretation of the data and helped to draft the manuscript. KD-D critically revised the manuscript. BK-C participated in the design of the study and statistical analyses. JK-S conceived the study, participated in its design and coordination, and drafted the manuscript. All authors read and approved the final manuscript.

## Conflict of Interest Statement

The authors declare that the research was conducted in the absence of any commercial or financial relationships that could be construed as a potential conflict of interest.
